# Analytic calculations of anharmonic infrared and Raman vibrational spectra

**DOI:** 10.1039/c5cp06657c

**Published:** 2016-01-07

**Authors:** Yann Cornaton, Magnus Ringholm, Orian Louant, Kenneth Ruud

**Affiliations:** a Centre for Theoretical and Computational Chemistry , Department of Chemistry , University of Tromsø—The Arctic University of Norway , N-9037 Tromsø , Norway . Email: yann.cornaton@uit.no ; Email: magnus.ringholm@uit.no ; Tel: +47 77623101; b Laboratory of Theoretical Chemistry , UCPTS , University of Namur , Rue de Bruxelles 61 , B-5000 Namur , Belgium

## Abstract

Using a recently developed recursive scheme for the calculation of high-order geometric derivatives of frequency-dependent molecular properties [Ringholm *et al.*, *J. Comp. Chem.*, 2014, **35**, 622], we present the first analytic calculations of anharmonic infrared (IR) and Raman spectra including anharmonicity both in the vibrational frequencies and in the IR and Raman intensities. In the case of anharmonic corrections to the Raman intensities, this involves the calculation of fifth-order energy derivatives—that is, the third-order geometric derivatives of the frequency-dependent polarizability. The approach is applicable to both Hartree–Fock and Kohn–Sham density functional theory. Using generalized vibrational perturbation theory to second order, we have calculated the anharmonic infrared and Raman spectra of the non- and partially deuterated isotopomers of nitromethane, where the inclusion of anharmonic effects introduces combination and overtone bands that are observed in the experimental spectra. For the major features of the spectra, the inclusion of anharmonicities in the calculation of the vibrational frequencies is more important than anharmonic effects in the calculated infrared and Raman intensities. Using methanimine as a trial system, we demonstrate that the analytic approach avoids errors in the calculated spectra that may arise if numerical differentiation schemes are used.

## Abstract 2

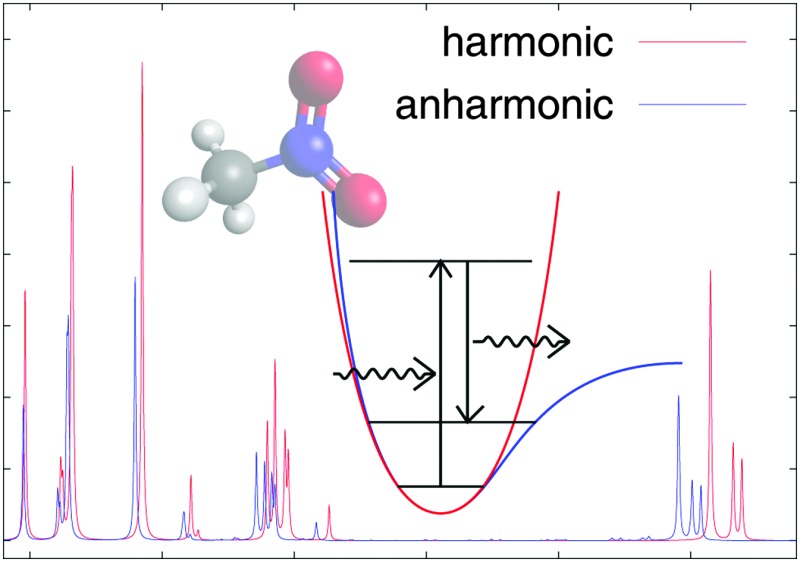
Using a recent recursive scheme for the calculation of high-order geometric derivatives of molecular properties, we present the first analytic calculations of infrared and Raman spectra including anharmonicity both in the vibrational frequencies and in the IR and Raman intensities.

## 1 Introduction

The calculation of spectroscopic phenomena involving molecular vibrations is an example of a successful application of theoretical chemistry to aid the interpretation of experimental observations. The calculation of molecular structure and vibrational spectra was made possible by the pioneering work of Bratoz, Pulay, Pople, Schaefer and others in developing methods for calculating analytical geometric derivatives of the molecular energy.^1–5^ Second-order geometric derivatives of the energy have since been derived and implemented for a wide range of correlated wave functions,^3,5–13^ as well as at the level of density functional theory (DFT).^14–16^ For a detailed historical account we refer to recent reviews of molecular properties in general and molecular force fields in particular.^17–19^

A commonly used approximation in the study of vibrational spectroscopies is the double-harmonic approximation,^20^ where molecular vibrations are described as harmonic oscillators and where the fundamental properties describing the spectroscopic intensities are determined by the first-order geometric derivative of the polarization property governing the spectroscopic phenomenon under study. This corresponds to a description which obeys the well-known selection rules for *e.g.* infrared (IR) and Raman spectroscopies,^21^ where it is the magnitude of the first-order geometric derivative of the molecular electric dipole moment and polarizability, respectively, that determines the spectroscopic intensity associated with the excitation to a singly excited state of a particular normal mode. Although coupled-cluster theory can provide vibrational frequencies of high accuracy,^22–28^ its computational cost prevents its routine use for larger molecules. For this reason, density-functional theory (DFT) has been gaining increasing popularity in recent years and has been used for calculations of Raman spectra of molecules as large as buckminsterfullerene.^29^ The calculations have often been done in combination with scaling of the frequencies in order to account for anharmonicities and errors inherent in the exchange–correlation functional used.^30,31^ A typical computational protocol has been to determine the frequencies of the (harmonic) normal modes by DFT using the B3LYP functional coupled with intensity calculations performed at the HF or DFT level,^32–34^ the choice of level of theory for the intensity calculations depending on the computational tools available for the calculation of the necessary geometric derivative of the pertinent polarization property.

By leaving the double harmonic approximation, it is possible to obtain a more accurate description of both the vibrational frequencies and the spectroscopic intensities.^23,35–38^ For the latter, the introduction of anharmonic effects will enable both an improved description of the intensities associated with a single excitation of a particular normal mode as well as introduce the leading-order contributions to intensities associated with transitions corresponding to the simultaneous excitation of two or more vibrational quanta, either involving only a single normal mode or several of them, often referred to as overtone and combination bands, respectively.

Calculations of anharmonic contributions for the purpose of correcting vibrational frequencies have regularly been carried out,^17,23,39–41^ requiring at least third-order geometric derivatives of the molecular energy, from here on referred to as the cubic force constants. We will refer to the corresponding fourth-order derivatives as the quartic force constants. Calculations of the cubic and quartic force constants have previously almost without exception been done using numerical differentiation.^23,41–43^ The only exception is the analytic calculation of cubic and quartic force constants at the HF level reported by Handy and coworkers.^44,45^ Recently, we presented an analytic implementation of cubic and quartic force constants at the DFT level^46^ by the use of a newly developed recursive code^47^ for the calculation of molecular properties by response theory.^48^

For the IR and (regular) Raman spectroscopies, programs that allow for the analytic calculation of the required first-order geometric derivatives of the dipole moment and polarizability, respectively, have been available for some time.^49–51^ The calculation of anharmonic corrections to the intensities in these spectroscopies requires both the development of the necessary vibrational perturbation theory^38^ to obtain expressions for these corrections and the possibility of calculating second- and third-order geometric polarization property derivatives, as well as the cubic and quartic force constants, that enter into these expressions. Programs that would allow for the analytic calculation of some of these properties are available, but such calculations have mainly been restricted to the HF level of theory, and for some of the properties (and more so if a DFT description is desired), the researcher has had to resort to numerical differentiation. Analytic calculation offers several advantages over numerical methods such as higher attainable accuracy and ease of computation,^51^ as numerical derivatives are sensitive to the finite perturbation/geometry displacements employed, and this can have significant effects on the results if not managed carefully.^52–54^ For these reasons, analytic methods are preferred over numerical ones.

In this work, we present the first application of our recursive approach for the analytic calculation of the anharmonic vibrational frequencies and infrared and Raman intensities of methanimine as well as nitromethane and its mono- and di-deuterated isotopomers. Methanimine has been shown to be very sensitive to the numerical differentiation parameters^52^ and thus provides a good illustration of the advantages of the analytic approach. The nitromethane isotopomers have been selected because experimental spectra display a large number of combination and overtone bands, for which calculation calls for the use of an anharmonic treatment. We remark that anharmonic effects have also been found to contribute appreciably to the spectroscopic intensities for several other molecules.^38^

The rest of the paper is organized as follows: in Section 2, we outline the theoretical foundation for the analytic calculation of anharmonic corrections to vibrational frequencies and IR and Raman intensities. In Section 3, we provide details about the computational setup used for the calculations on our chosen systems. We present and discuss the results of our calculations in Section 4, and make some concluding remarks in Section 5.

## 2 Theory

We will begin in Section 2.1 by outlining how the high-order molecular properties used in this work can be calculated analytically through the use of our recently developed recursive response code and then in Section 2.2 proceed to show how these properties can be used to determine anharmonic corrections to vibrational frequencies and IR and Raman intensities. Although the general framework has been described previously,^46–48^ this work is the first report of fifth-order analytic derivatives involving geometrical distortions.

### 2.1 Analytic calculation of response properties

A detailed presentation of the response theory, which in our approach is fundamental for the analytic calculation of the cubic and quartic force fields and the high-order geometric derivatives of the dipole moment and polarizability that are needed in this work, is too long to show here, and we will therefore restrict ourselves to the most salient features. We refer to the original work^48^ for a more thorough treatment, and to our recent work^47^ for a description of the recursive implementation used in the present work.

Our analytic scheme uses as a starting point that linear response functions described by perturbations *a* and *b* can be formulated as perturbation strength *ε*_*i*_ (*i* = *a*, *b*,…) derivatives of a quasienergy Lagrangian gradient, expressed in a density-matrix (**D**) formulation as^48^
1
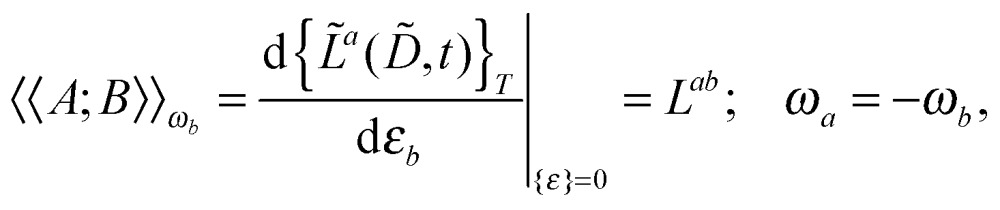
where the derivative is evaluated at zero perturbation strength and where higher-order response functions can be found by further differentiation of eqn (1). A tilde is used to represent a quantity considered at an arbitrary perturbation strength, and the absence of a tilde denotes evaluation at zero perturbation strength. The quasienergy Lagrangian *L* ^*a*^ is given by2
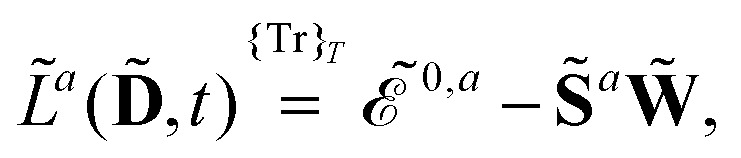
where we have introduced the atomic orbital (AO) overlap matrix **S** as

3 *S*_*μν*_ = *χ̃*_*μ*_|*χ̃*_*ν*_,

where *χ̃* is an atomic orbital, and where the energy- and frequency-weighted Fock matrix **W** is defined as4
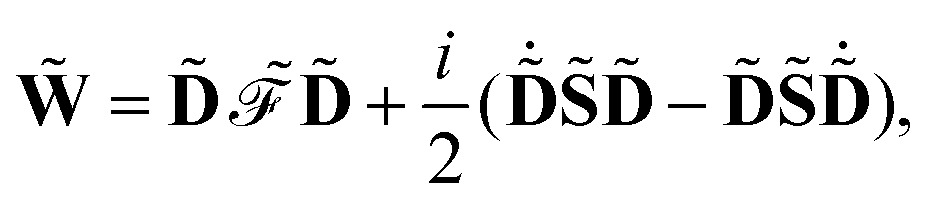
where the generalized Kohn–Sham Fock matrix 
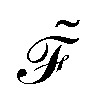
 is given by5
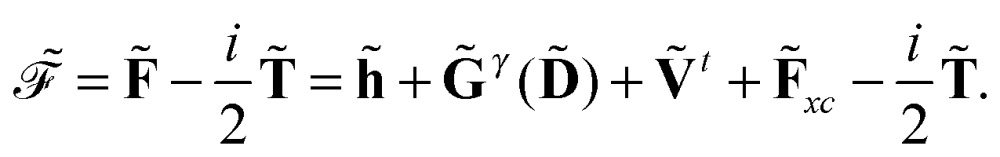
 We also introduced the generalized energy 
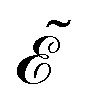
 as6
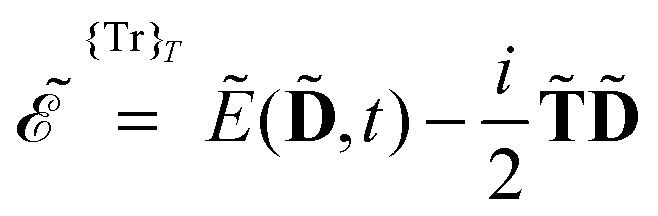

7
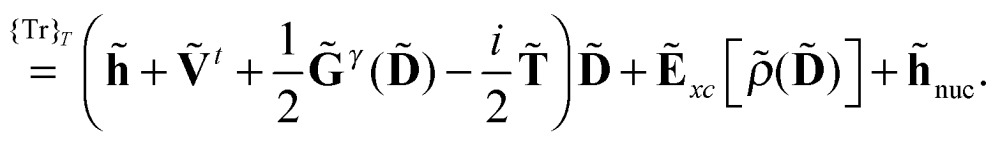
 In eqn (5)–(7), we introduced the half-time-differentiated overlap matrix **T**, the one-electron matrix **h**, the external field operator **** ^*t*^ and the two-electron matrix **G** ^γ^ with γ-fractional exchange as8
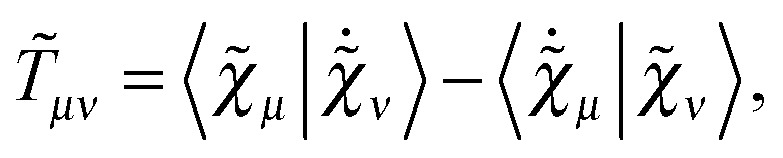

9
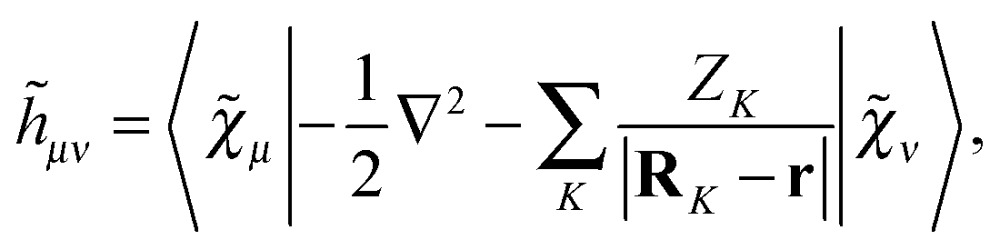

10
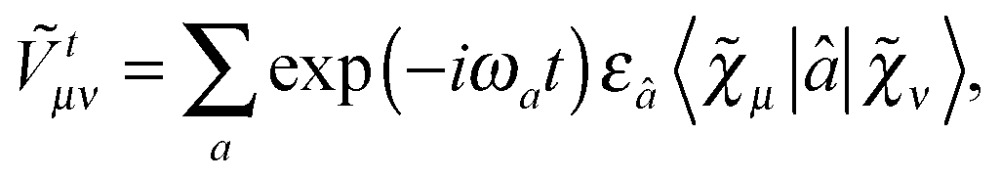

11
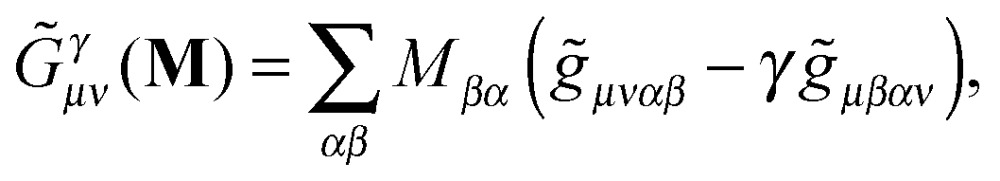
and also the exchange–correlation contributions **F**_*xc*_ and **_*xc*_[*ρ̃*(**D**)] in addition to a nuclear potential operator *h*_nuc_. Here and throughout the paper, atomic units are used unless otherwise stated. Molecular properties characterized by a perturbation tuple *abc*… can therefore be formulated as derivatives of the quasienergy Lagrangian gradient as12
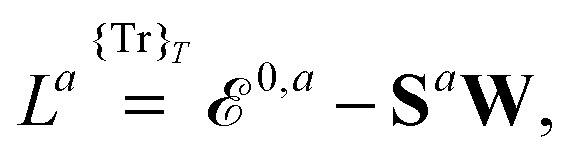

13
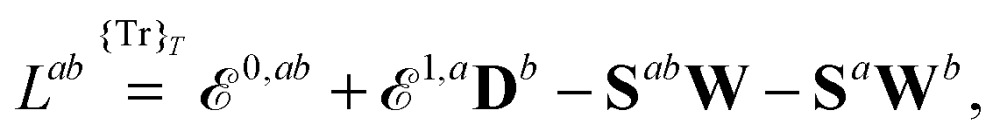

14
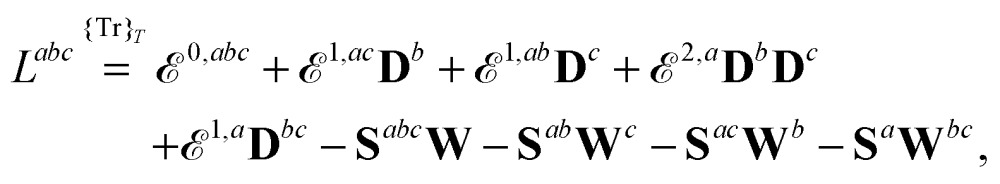
where we have introduced a short-hand notation for differentiation and tracing by15
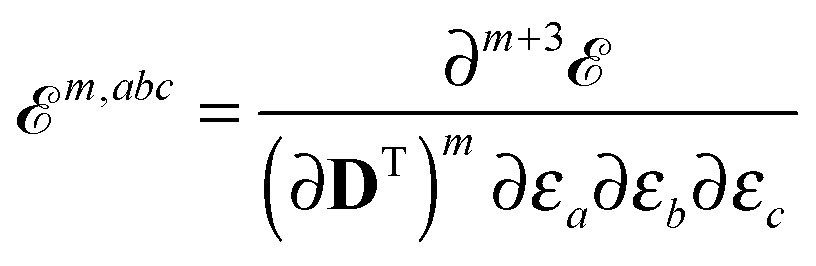
and16
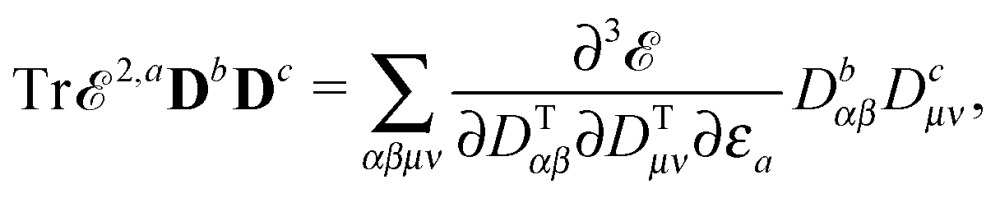
respectively. This theory is sufficient to define any response function using the so-called *n* + 1 rule formulation,^55^ where the calculation of a response property of order *n* + 1 requires the calculation of the density matrix perturbed to order *n*. However, other formulations placing other conditions on which perturbed density (and Fock) matrices must be calculated are possible.^55^ Let us represent the idempotency of the density matrix and the time-dependent self-consistent field (TDSCF) conditions as the matrices **Y** and **Z**, respectively, so that

17 **Y** = **DSD** – **D**,

and18
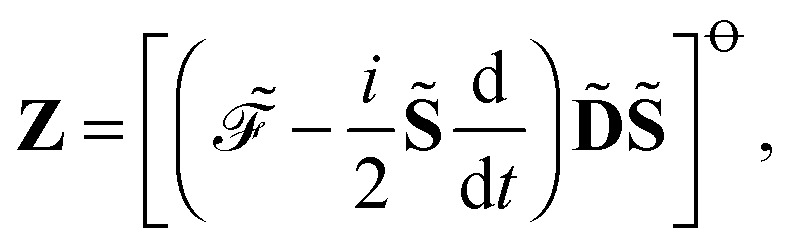
where the notation

19 [**M**]^⊖^ = **M** – **M**^†^,

and

20 [**M**]^⊕^ = **M** + **M**^†^,

has been introduced, and where adjungation is defined to happen before time differentiation. It can be shown that the ansatz

21 *λ̃*_*a*_ = [**D**^*a*^**SD**]^⊖^,

for the multiplier *λ̃*_*a*_ for **Y** leads to the definition of the multiplier *ζ̃*_*a*_ for **Z** as22
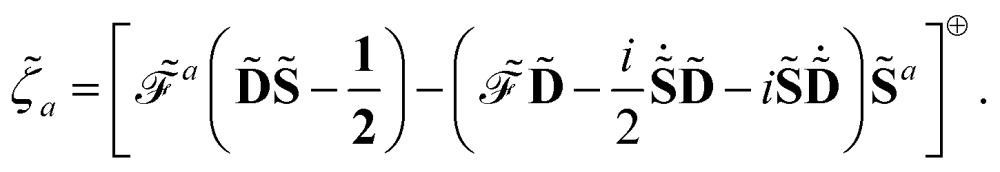
 It is then possible to make a general expression for the quasi-energy Lagrangian for the calculation of response properties as23
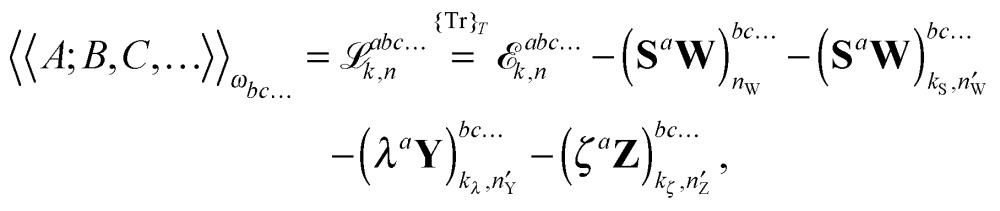
where the values of *k* and *n* in the various terms denote, with minor variations, to which orders perturbed Fock and density matrices must be calculated in order to evaluate this expression: the value of *k* determines to which order the perturbed matrices must be calculated for perturbation tuples involving perturbation *a*, whereas the value of *n* determines the same for perturbation tuples not involving perturbation *a*. We have that *k* + *n* = *N* – 1, where *N* is the order of the property considered, and *k* must be chosen as an integer in the interval *k* ∈ [0,(*N* – 1)/2], where (*N* – 1)/2 is rounded down for even *N*. In this work, we do not discuss how the necessary perturbed Fock and density matrices can be calculated, as it is described in detail in ref. 48. We remark, however, that since the calculation of high-order properties requires solving linear response equation systems and since this part of the calculation is computationally expensive, a judicious (*k*,*n*) rule choice may give a significant reduction in the number of such systems to be solved, both compared to other rule choices and to numerical differentiation schemes. For instance, for the calculation of cubic force constants, the choice (*k*,*n*) = (1,1) makes it necessary to solve *M* systems, where *M* is the number of geometrical coordinates, whereas (*k*,*n*) = (0,2) results in *M* ^2^ such systems. Similarly, a scheme where an analytically calculated molecular Hessian is differentiated numerically by nuclear displacements results in the number of such systems being of the order of *M* ^2^. Similar savings can be achieved for other properties.

With the recursive program developed by our group,^47^ it is possible to evaluate eqn (23) for any response property, including the calculation of the required perturbed Fock and density matrices, as long as external routines are available that can provide the necessary (un)perturbed one- and two-electron integral contributions,^56–58^ exchange–correlation contributions^59,60^ and perturbed nuclear potential contributions, and solve the response equations^61,62^ that arise during the evaluation of perturbed Fock and density matrices. More information about the external modules used in this work is given in Section 3. All such modules used in the present work have been parallelized; see *e.g.* ref. 63.

### 2.2 Anharmonic corrections to vibrational frequencies and spectroscopic intensities

Having determined the harmonic vibrational frequencies and normal modes of vibration from the well-established eigenanalysis of the molecular Hessian,^20^ it is possible to make anharmonic corrections to fundamental vibrational frequencies and frequencies corresponding to combination or overtone excitations of the normal modes by a second-order perturbational approach, where the resulting expressions involve the cubic and quartic force constants and Coriolis vibration–rotation coupling constants. In the VPT2 approach,^23,35,36^ the corrected fundamental vibrational frequencies *ν*_*i*^1^_, first overtone frequencies *ν*_*i*^2^_ and first combination frequencies *ν*_*i*^1^*j*^1^_ are given as, respectively24
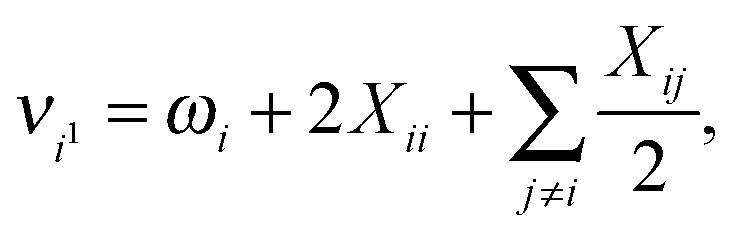

25 *ν*_*i*^2^_ = 2*ν*_*i*^1^_ + 2*X*_*ii*_,

26 *ν*_*i*^1^*j*^1^_ = *ν*_*i*^1^_ + *ν*_*j*^1^_ + *X*_*ij*_,

where the diagonal and off-diagonal correction terms *X*_*ii*_ and *X*_*ij*_ are given by27
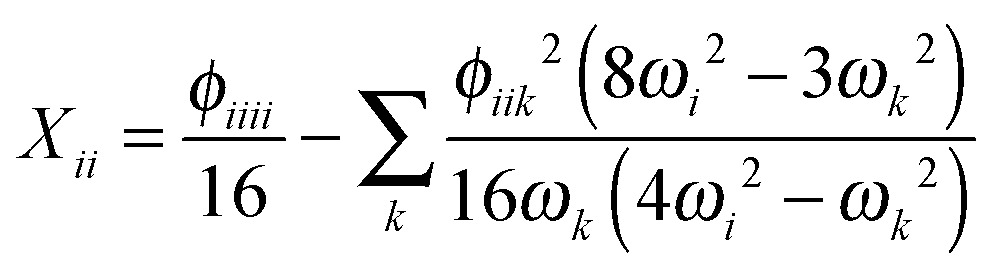
and28
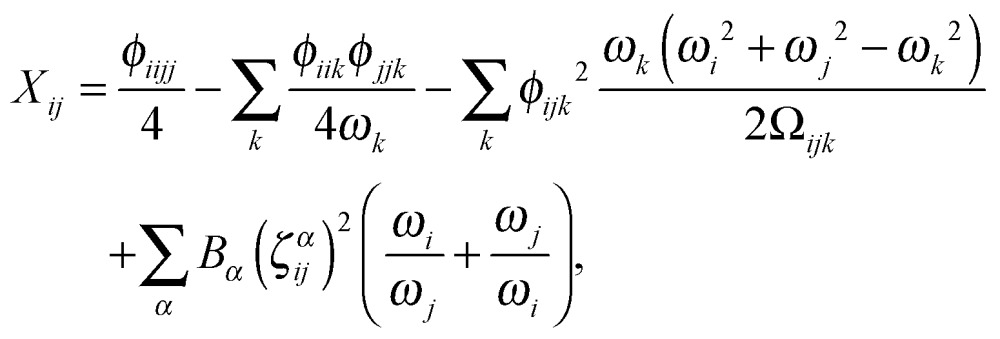
where *Ω*_*ijk*_ is defined as

29 *Ω*_*ijk*_ = (*ω*_*i*_ + *ω*_*j*_ + *ω*_*k*_)·(–*ω*_*i*_ + *ω*_*j*_ + *ω*_*k*_)·(*ω*_*i*_ – *ω*_*j*_ + *ω*_*k*_)·(*ω*_*i*_ + *ω*_*j*_ – *ω*_*k*_).

In the above expressions, *ω*_*i*_ denotes a harmonic fundamental frequency, *φ*_*ijk*_ and *φ*_*ijkl*_ are cubic and quartic force constants, respectively, *B*_*α*_ is the rotational constant for axis *α*, and *ζ* *α* *ij* is a Coriolis coupling constant.

The method chosen in the present work is the so-called generalized vibrational second-order perturbation (GVPT2) model.^38,41^ In this method, a VPT2 treatment of the molecular vibrations is used, except for the cases where Fermi resonances are considered to have occurred. In these cases, the terms in the VPT2 treatment that are affected by the Fermi resonance are not included,^23^ and the affected frequencies are instead resolved in a variational approach.

Expressions for corrections to spectroscopic intensities can also be identified by a perturbation-theory approach. In a recent work by Bloino and Barone,^38^ GVPT2 expressions for IR and Raman intensities have been derived. The expression for the IR intensities is30
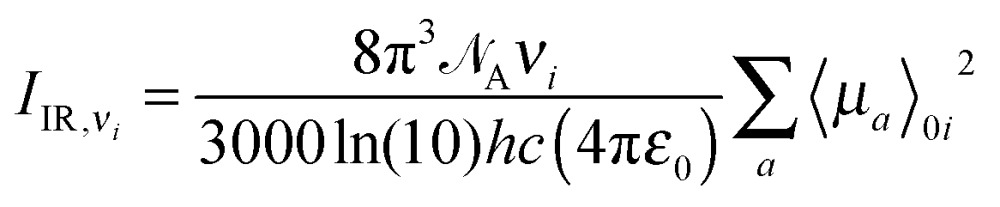
and in classical Raman spectroscopic measurements, the unpolarized (as well as polarized) scattering intensity at a temperature *T*, related to the Raman cross section, is given by31
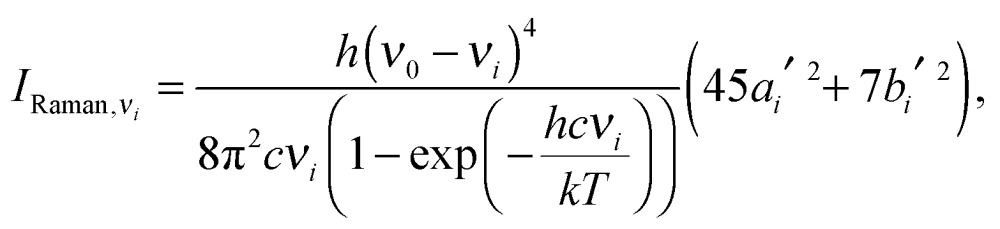
where32
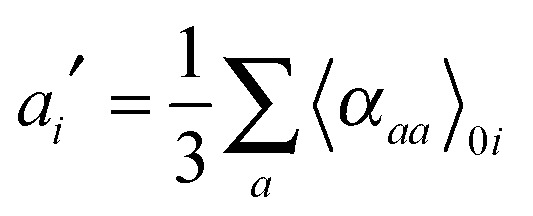
and33
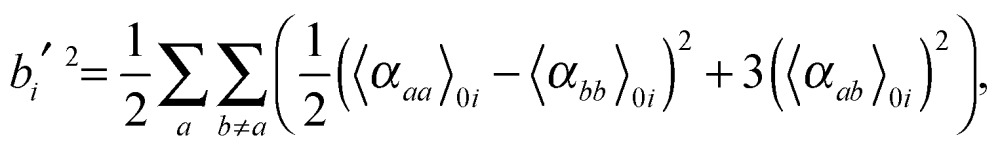
where *ν*_*i*_ = *ω*_*i*_ in the harmonic approximation and is given by eqn (24)–(26) in the anharmonic GVPT2 treatment, *ν*_0_ is the frequency of the incident laser in the Raman experiment, and  _0*i*_ represents the transition moment of the relevant polarization property from the vibrational ground state to the *i*th vibrational excited state. In the double-harmonic treatment, these transition moments are determined by first-order geometric derivatives of the polarization property (*P*_0*i*^1^_ = ∂*P*/∂*q*_*i*_), whereas the anharmonic expressions also involve the second- and third-order geometric derivatives of the polarization property and the cubic and quartic force constants. The resulting expressions in the anharmonic case are large and we refer to the work of Bloino and Barone^38^ where the complete expressions are reported.

Altogether, the expressions used in the complete VPT2 treatment involve the first-, second-, and third-order geometric derivatives of the molecular electric dipole moment and polarizability in the IR and Raman case, respectively, in addition to the cubic and quartic force constants, meaning that the highest-order property that must be calculated, *i.e.* the cubic force constants of the frequency-dependent polarizability, is a fifth-order energy derivative. The contributions to this property can be identified from eqn (23) and are shown here in order to demonstrate the complexity involved in the analytic calculations performed in this work and to justify the use of a recursive approach.

The third-order geometric derivative of the polarizability can be defined from a perturbation tuple (*a*, *b*, *c*, *d*, *e*), where perturbations *a*, *b* and *c* correspond to differentiation with respect to geometrical displacements, and *d* and *e* to differentiation with respect to a frequency-dependent electric dipole perturbation. Denoting a geometric perturbation as *g* and the two electric dipole perturbations as *f*_ω_ and *f*_–ω_, where, respectively, each perturbation is associated with a positive or negative frequency *ω*, eqn (23) takes the form34
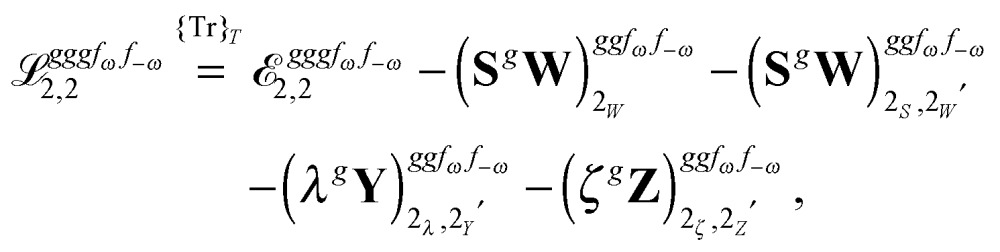
where the rule choice (*k*,*n*) = (2,2) is used because this will give the lowest computational cost. Omitting terms that must be zero straightforwardly or because, in the differentiation carried out, there was lack of dependence on the perturbation operators and, for the sake of brevity, writing contributions that are permutations of identical operators only once, the terms in eqn (34) can be written as35
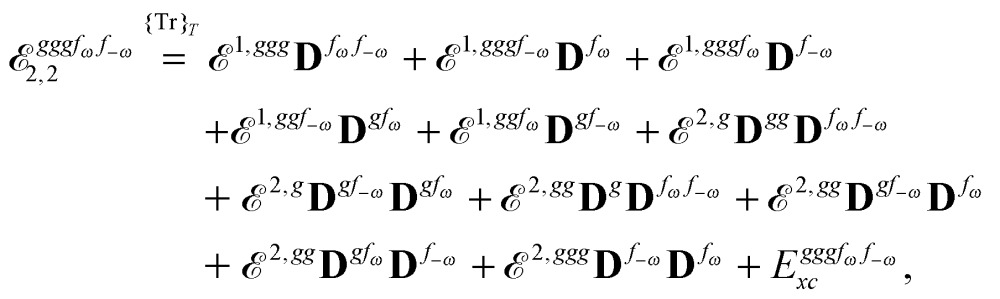

36
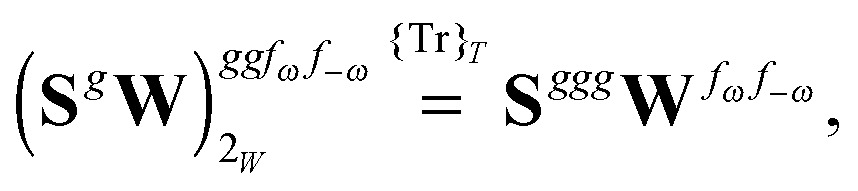

37
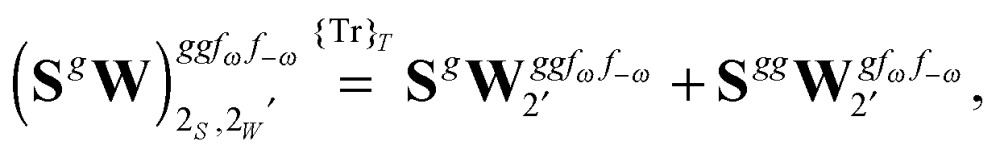

38
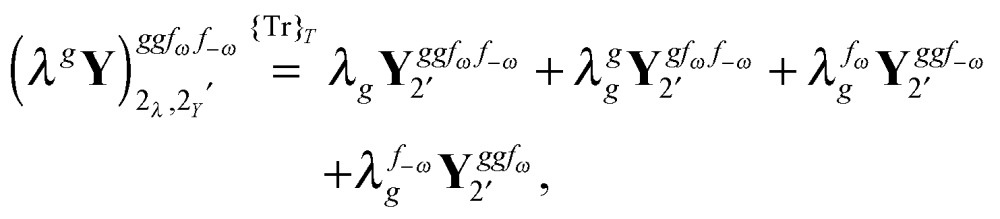
and39
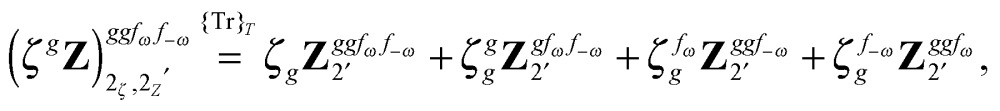
where, for example, 
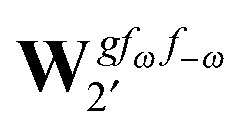
 from eqn (37) is40
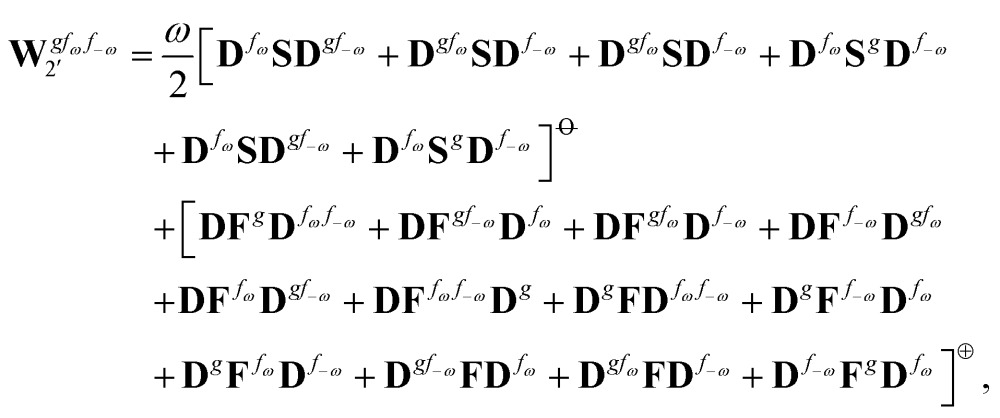
and where the other differentiated **W**, **Y**, and **Z** terms are of a similar complexity. We consider the length of these expressions, in particular eqn (40), and the corresponding complexity in treating them, as strongly supporting the use of a recursive approach for calculations of the high-order properties required for the GVPT2 treatment, and in a similar manner, automated approaches based on automatic differentiation are needed in order to evaluate the differentiated exchange–correlation energy and kernel 
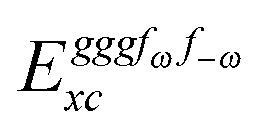
.^59^

## 3 Computational details

To compute the cubic and quartic force constants and the first-, second- and third-order geometric derivative tensors of the electric dipole moment and of the electric dipole polarizability, the recursive implementation^47^ of the open-ended response theory framework of Thorvaldsen *et al.* ^48^ has been used. This formalism has been implemented in a development version of the Dalton2013 program package.^64,65^ The linear response solver of Jørgensen *et al.* ^61^ has been used for the solution of the response equations. Differentiated one- and two-electron integrals were computed using the Gen1Int^56,57^ and Cgto-Diff-Eri^58,66^ programs, respectively, except for some of the lower-order two-electron integral geometric derivatives which were computed using existing functionality in Dalton. The differentiated exchange–correlation (XC) energy and potential contributions up to fifth order needed in the DFT calculations were computed using the XCFun library,^59,60^ where the integrator XCInt has been used for the integration of the XC contributions. The calculation of the Coriolis coupling constants is not done in a response theory framework, but have been calculated in the manner outlined in ref. 67.

**Fig. 1:**
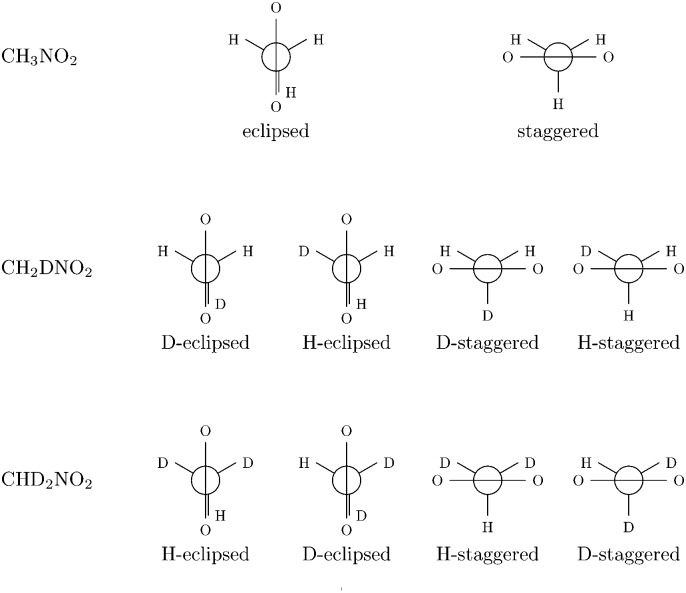
Newman projection of the different conformations of nitromethane considered.

All calculations have been performed at the DFT level of theory using the B3LYP hybrid functional.^68–70^ This functional has already been shown to give good results for the calculation of higher-order properties in earlier work.^46,71^ Dunning's correlation-consistent polarized triple-ζ (cc-pVTZ) basis set^72^ has been used. The study was conducted for methanimine (CH_2_NH), and nitromethane (CH_3_NO_2_) and its mono- (CH_2_DNO_2_) and di-deuterated (CHD_2_NO_2_) isotopomers. Two conformations (eclipsed and staggered) have been considered for the non-deuterated isotopomer and four (H-eclipsed, D-eclipsed, H-staggered and D-staggered) for each deuterated isotopomer (*cf.* Fig. 1).

For each system, the geometry was optimized and the molecular Hessian and the rotational constants were computed using the Dalton2013 program package.^64,65^ The other relevant molecular properties were computed at the optimized geometry using the recursive response property implementation, and the Coriolis coupling constants have been implemented in a development version of Dalton2013. The molecular Hessian was then used in a vibrational analysis to find the harmonic vibrational frequencies and to transform the geometric differentiation in the property tensors from a Cartesian basis to a reduced normal coordinate basis^73^ to calculate anharmonic frequencies and spectral intensities.

Anharmonic corrections to the fundamental frequencies, as well as first overtones and combination band frequencies were calculated from the cubic and quartic force constants, the rotational constants and the Coriolis coupling constants using a scheme based on vibrational second-order perturbation theory^35,36^ as described in Section 2.2, where terms found to be affected by Fermi resonances are taken out of the perturbational treatment^23^ and resolved variationally^41^ using the GVPT2 model.^38^

First-order geometric derivatives of the electric dipole and electric dipole polarizability in the reduced normal coordinate basis were used for the evaluation of the harmonic IR intensities and Raman scattering cross-sections, respectively. Anharmonic corrections to the spectral intensities were calculated by further considering the second and third geometric derivatives of the corresponding properties and the cubic and quartic force constants, in a reduced normal coordinate basis, using the GVPT2 model, resulting in features associated with corrections to the fundamental bands and the appearance of the first overtone and combination bands.

For methanimine, the cubic and quartic force fields have also been evaluated by numerical differentiation from the molecular Hessians calculated for Cartesian displacements *δx* of 10^–2^ and 10^–3^ Å with Dalton2013 using the expressions41
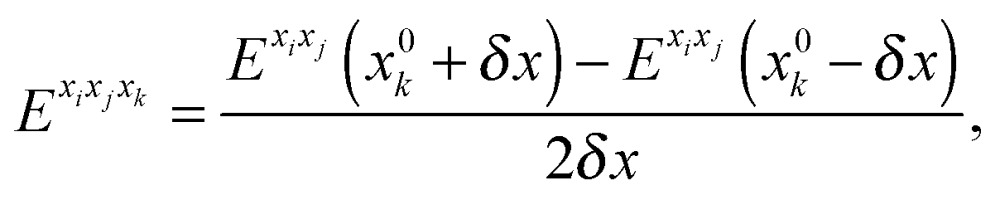

42
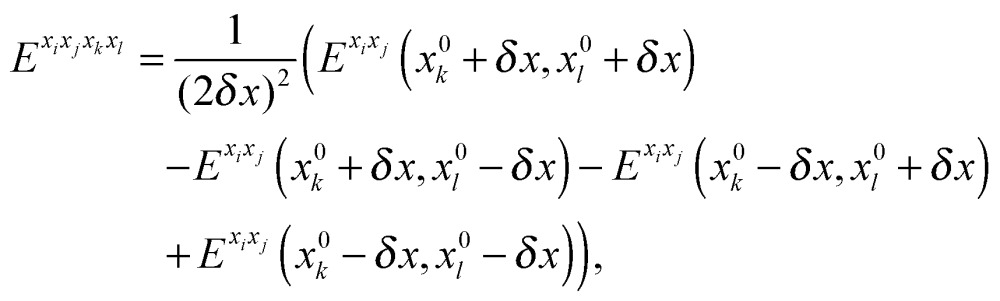
where *E* ^*x*_*i*_*x*_*j*_^, *E* ^*x*_*i*_*x*_*j*_*x*_*k*_^ and *E* ^*x*_*i*_*x*_*j*_*x*_*k*_*x*_*l*_^ represent, respectively, the second-, third- and fourth-order derivatives of the energy with respect to the Cartesian components in superscript, and using convergence thresholds of 10^–8^ for both the molecular orbital (MO) coefficients and relative to the norm of the perturbed MO coefficients when solving the response equations. The same convergence criteria have been applied to all fully analytic calculations. We remark that the errors in the calculated properties resulting from these strict thresholds are negligible.

The first, second and third geometry derivatives of the electric dipole moment and polarizability have also been evaluated this way and with the same convergence thresholds, but using the expressions43
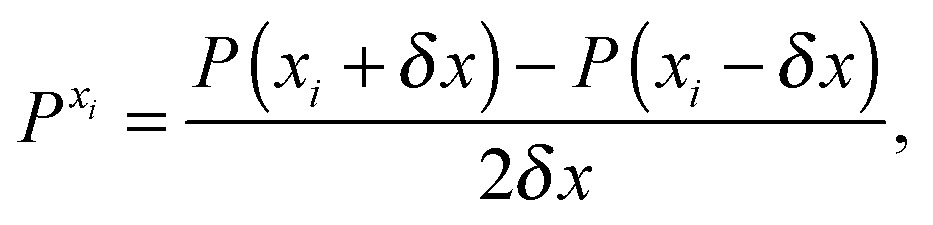

44
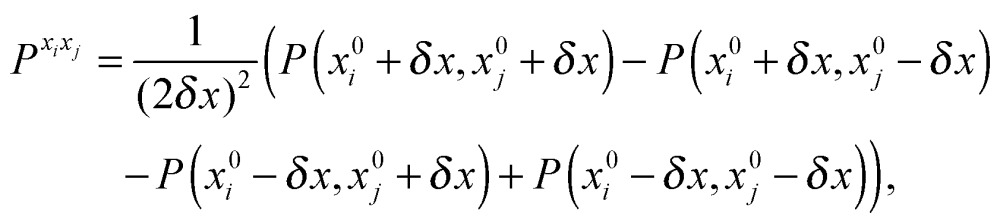

45
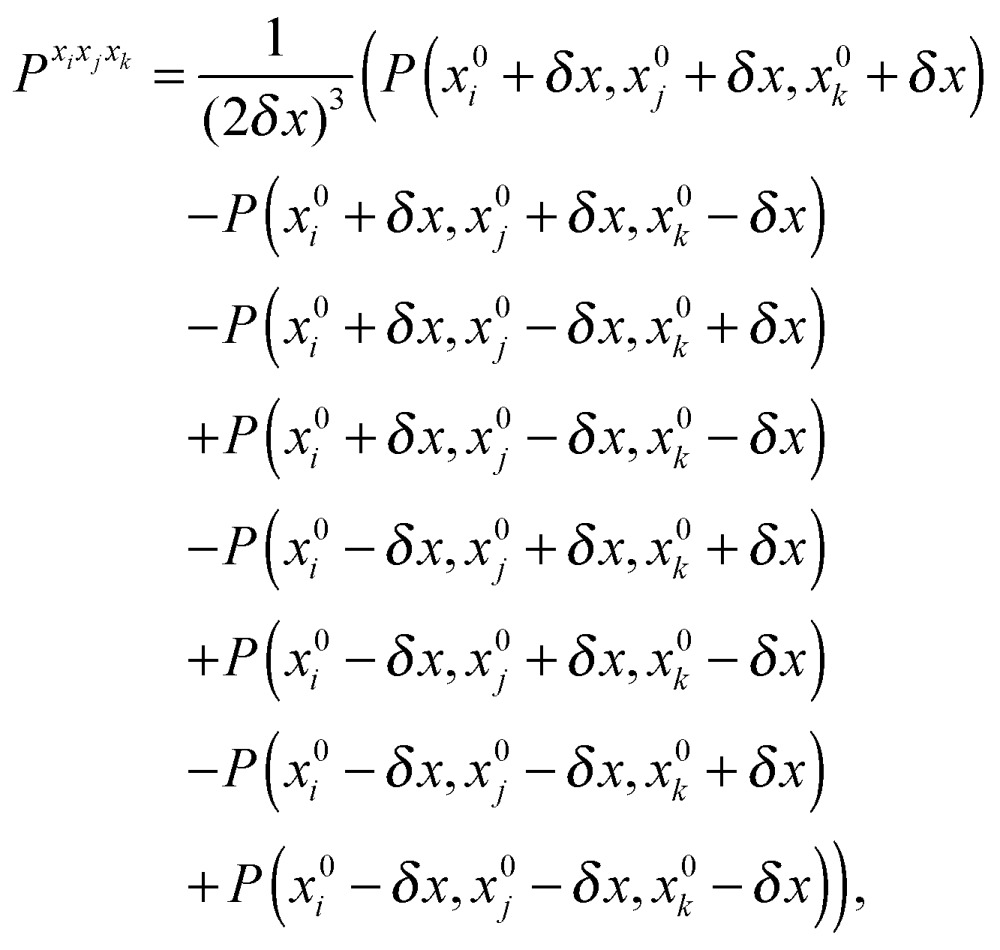
where *P* denotes either the electric dipole moment or the electric polarizability, and *P* ^*x*_*i*_^, *P* ^*x*_*i*_*x*_*j*_^ and *P* ^*x*_*i*_*x*_*j*_*x*_*k*_^ represent respectively the first, second and third derivatives with respect to geometry distortions.

The spectral bands have been modeled using Lorentzian functions for the band shape with a 10 cm^–1^ full width at half maximum. A 1 cm^–1^ resolution was used to plot all spectra. Raman spectra have been evaluated considering an incident laser wavelength of 514 nm, corresponding to an Ar^+^ laser at 298.15 K.

## 4 Results and discussion

### 4.1 Reliability of the approach: methanimine

In this section, we will illustrate the need for analytic differentiation techniques by calculating the infrared and Raman spectra of methanimine (CH_2_NH), comparing the analytic approach to the results obtained by numerical differentiation using different step lengths. The sensitivity of methanimine to numerical differentiation parameters^52^ makes it a suitable system for illustrating the advantages of using an analytic approach.

The theoretical vibrational frequencies obtained using the different approaches are compiled in Table 1. Experimental values^74,75^ are also presented for comparison.

**Table 1:** Calculated (harmonic, numerical anharmonic and analytic anharmonic) and experimental normal vibrational frequencies of CH_2_NH (in cm^–1^)

	Mode	*ω*_*i*_	*ν* num. *i* (10^–2^ Å)	*ν* num. *i* (10^–3^ Å)	*ν* anal. *i*	2*ν*expt*i* ^*a*^
1	*ν*(NH)	3424	3251	3250	3240	3263
2	*ν*_a_(CH_2_)	3100	2932	2922	2923	3024
3	*ν*_s_(CH_2_)	3007	2844	2827	2839	2914
4	*ν*(CN)	1712	1681	1685	1677	1638
5	*δ*(CH_2_)	1492	1465	1450	1463	1452
6	*δ*(HNC)	1373	1336	1303	1332	1344
7	*τ*(CH_2_)	1169	1138	1100	1135	1127
8	*ω*(CH_2_)	1101	1081	999	1078	1061
9	*ρ*(CH_2_)	1075	1063	1087	1062	1058

^*a*^Experimental data from ref. 74 and 75.

In the case of numerical differentiation using a step length of *δx* = 10^–3^ Å, the difference in Hessian values between some of the displaced systems was smaller than the numerical precision, thus illustrating one of the problems of this approach. This can be illustrated by the anharmonic correction to the vibrational frequency of the 9^1^ mode which is positive, whereas anharmonic corrections are generally expected to be negative, as is obtained in the analytic approach and when a step length of *δx* = 10^–2^ Å is used in the numerical differentiation approach. The anharmonic corrections to the vibrational frequencies of the high-frequency modes appear less sensitive to this problem. The analytic approach does not depend on the energy difference between slightly displaced systems and is therefore free from this source of numerical error.

Using a step length of *δx* = 10^–2^ Å for the numerical differentiation, numerical noise is largely avoided and the anharmonic frequencies are in better agreement with experimental fundamental frequencies. This is also observed for the anharmonic frequencies obtained by analytic differentiation. Nevertheless, the numeric anharmonic corrections are still on average in error by about 10% compared to the analytic corrections, the latter being always larger than the former.

**Fig. 2:**
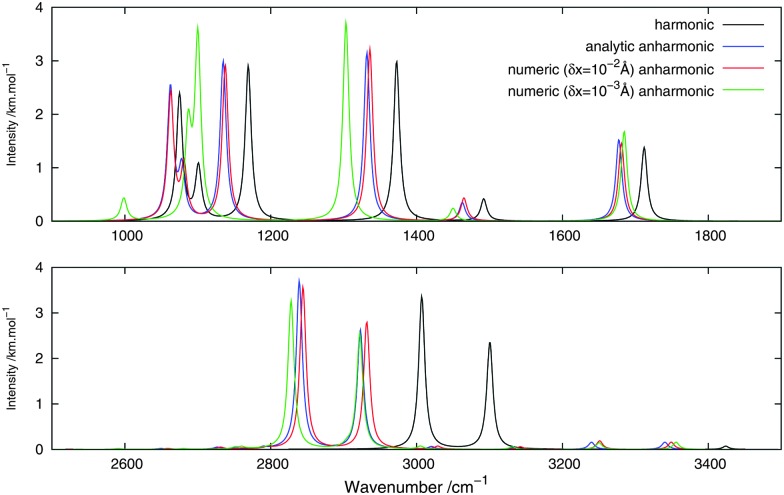
Theoretical infrared spectrum of CH_2_NH comparing different derivation approaches.

**Fig. 3:**
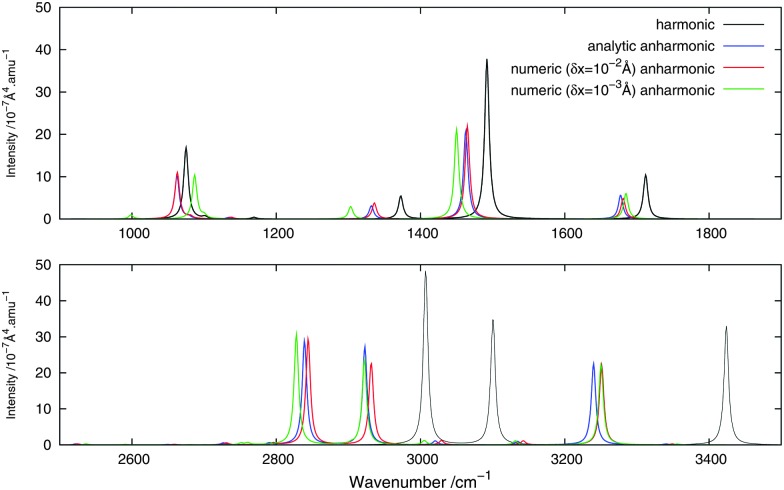
Theoretical Raman spectrum of CH_2_NH comparing different derivation approaches.

Fig. 2 and 3 show, respectively, the calculated infrared and Raman spectra of methanimine for the analytical and numerical approaches. In the calculated infrared spectrum, using a step length of *δx* = 10^–3^ Å in the numerical differentiation, not only are the anharmonic corrections to the frequencies in poor agreement with the analytic ones, but so are also the corrections to the intensities, most strikingly so for the low-frequency peaks. In this case, for the IR spectrum, the *δx* = 10^–3^ Å numerical differentiation reproduces the analytic anharmonic spectral intensities almost perfectly for the peaks of frequency above 2900 cm^–1^ but overestimates (in absolute value) drastically the intensity for the other peaks, the lower the frequency the larger the overestimation.

Numerical (*δx* = 10^–2^ Å) and analytic anharmonic corrections to the spectral intensities both go in the same direction for each individual peak, but the magnitude of the corrections differs. The difference in the intensity of the anharmonic corrections to the infrared intensities between the numerical and analytic values varies from 10 to 230% of the analytic correction depending on the peak considered, with the majority of the corrections being in error by 35–85%, the only exceptions being the low-energy modes 8^1^ and 9^1^. However, there is no trend as to whether the numerical corrections under- or overestimate the analytic results. As the anharmonic corrections to the total intensity of the peaks is small, these differences are not easily visible from the spectra plotted in Fig. 2.

For the Raman spectra, numerical noise does not affect the derivatives of the electronic polarizability when using a step length of *δx* = 10^–3^ Å. The numerical anharmonic corrections to the spectral intensities are thus in better agreement with the analytic ones than in the infrared case, but the corrections to the vibrational frequencies remain wrong. Considering the intensities, the numerical spectrum obtained using a step length of *δx* = 10^–3^ Å shows differences of less than 10% compared to the analytic spectrum, and is thus in better agreement than the spectrum obtained using a step length of *δx* = 10^–2^ Å, where these differences may be as large as 20%. The only exception is the 8^1^ mode, for which both step lengths give corrections that are far from the analytic one. As for the IR spectra, the calculated corrections can be both larger and smaller than the analytic result and whether the corrections are over- or underestimated also depends on the step length. It should also be noted that, depending on the step length used, the ordering of the intensity of the peaks can differ. For example, in the case of *δx* = 10^–2^ Å, 1^1^ is slightly more intense than 2^1^, whereas with *δx* = 10^–3^ Å, the 2^1^ peak is more intense than 1^1^, in agreement with the analytic differentiation results.

This example illustrates that even if the use of numerical differentiation can lead to qualitatively sound results, it still depends strongly on the step length used. While methanimine is still a rather small molecule, it could still be expected that these difficulties will be present in larger systems. On this note, we now turn our attention to using the analytic approach to calculate anharmonic vibrational spectra and compare these with available experimental observations.

### 4.2 Comparison with experiment: nitromethane

In Section 4.2.1, we will present and discuss the computed vibrational frequencies, before we in Sections 4.2.2 and 4.2.3 turn to a discussion of the theoretical IR and Raman spectra, respectively, comparing our theoretical results to available experimental data.

All experimental and theoretical studies^76–79^ on the geometry of nitromethane agree that the barrier (Δ*E* = 9.6*μE*_h_^76^) for the rotation of the methyl group around the CN axis is very small, with the staggered conformation being slightly more stable. Our results reproduce quantitatively the barrier height (Δ*E*_B3LYP_ = 10*μE*_h_). Such a low barrier makes it necessary to consider several rotamers when modeling the theoretical spectra, and for this reason all the geometries corresponding to the extrema of the energy along the rotation of the methyl group are considered in this study (*cf.* Fig. 1). A Boltzmann averaging at room temperature of these rotamers would give a quasi-equal weight for each of the conformers, and for this reason all rotamers will thus be considered of equal weight in the averaging of the spectra from the different rotamers. We note that such a treatment for the low-frequency internal rotation of the methyl group has to be considered approximate, and that this vibration mode probably should be treated by a non-local representation going beyond the normal-mode approximation. For this reason, we will in the following not include this mode in the anharmonic treatment.

#### 4.2.1 Vibrational frequencies

For all rotamers of each isotopomer, the frequency corresponding to the rotation of the methyl group is found to be quite small at the harmonic level and negative at the anharmonic level, which is consistent with what can be expected for a quasi-free rotating methyl group.^76,77^ The observed spectra should therefore come from the average over all the rotamers. For this study, only the extremum rotamers (staggered and eclipsed) have been considered (*cf.* Fig. 1), and the system has been treated as having only 14 normal modes (instead of 3*N* – 6 = 15) by not considering the derivatives with respect to the methyl rotation mode in the anharmonic calculations.

Using partially deuterated isotopomers lowers the symmetry of the system, thus allowing new rotamers to be spectroscopically active and giving rise to band splittings.^77,78^ Calculated (harmonic and anharmonic) frequencies for the fundamentals of the non-, mono- and di-deuterated isotopomers of nitromethane are compiled in Tables 2, 3 and 4, respectively. Experimental frequencies^77,79–81^ are also given for comparison.

**Table 2:** Calculated (harmonic and anharmonic) and experimental normal vibrational frequencies of CH_3_NO_2_ (in cm^–1^)

Mode		Eclipsed		Staggered		Expt.^*a*^
		*ω*_*i*_	*ν*_*i*_	*ω*_*i*_	*ν*_*i*_	*ν*_*i*_
1	*ν*_a_(CH_3_)	3194	3039	3193	3038	3080
2	*ν*_s_′(CH_3_)	3161	3005	3161	3006	3045
3	*ν*_s_(CH_3_)	3076	2956	3074	2953	2974
4	*ν*_a_(NO_2_)	1632	1583	1632	1584	1583
5	*δ*_s_′(CH_3_)	1477	1427	1478	1427	1434
6	*δ*_a_(CH_3_)	1466	1416	1464	1414	1410
7	*ν*_s_(NO_2_)	1427	1388	1427	1388	1397
8	*δ*_s_(CH_3_)	1398	1356	1399	1358	1380
9	*ρ*_⊥_(CH_3_)	1136	1107	1136	1106	1131
10	*ρ*_∥_(CH_3_)	1109	1081	1109	1084	1096
11	*ν*(CN)	925	898	924	898	918
12	*δ*(NO_2_)	657	640	662	645	657
13	*ω*(NO_2_)	624	614	616	605	603
14	*ρ*(NO_2_)	482	475	481	475	475

^*a*^Experimental data from ref. 79 and 80.

**Table 3:** Calculated (harmonic and anharmonic) and experimental normal vibrational frequencies of CH_2_DNO_2_ (in cm^–1^)

Mode		D-eclipsed		H-eclipsed		D-staggered		H-staggered		Expt.^*a*^
		*ω*_*i*_	*ν*_*i*_	*ω*_*i*_	*ν*_*i*_	*ω*_*i*_	*ν*_*i*_	*ω*_*i*_	*ν*_*i*_	*ν*_*i*_
1	*ν*_a_(CH_2_)	3160	3003	3188	3033	3193	3037	3173	3018	3071
2	*ν*_s_(CH_2_)	3108	2971	3113	2975	3121	2985	3104	2965	3002
3	*ν*_∥_(CD)	2321	2249					2313	2244	2276
3	*ν*_m_(CD)			2294	2197	2283	2191			2266
4	*ν*_a_(NO_2_)	1626	1578	1630	1581	1631	1581	1627	1579	1578
5	*δ*(CH_2_)	1452	1407	1459	1413	1462	1413	1454	1408	1426
6	*ν*_s_(NO_2_)	1415	1372	1421	1377	1420	1376	1418	1375	1387
7	*ω*(CH_2_)	1318	1286	1311	1280	1310	1271	1315	1283	1288
8	*ρ*(CH_2_)	1303	1267	1287	1250	1285	1249	1296	1260	1258
9	*δ*_⊥_(CD)	1084	1054	1082	1055	1080	1054	1084	1055	1068
10	*δ*∥(CD)	945	918	976	951	988	963	955	929	957
11	*ν*(CN)	900	881	911	889	916	892	904	883	898
12	*δ*(NO_2_)	645	628	651	635	661	645	645	629	651
13	*ω*(NO_2_)	616	605	586	576	561	551	607	596	579
14	*ρ*(NO_2_)	455	449	466	460	476	470	456	450	454

^*a*^Experimental data from ref. 79, 80 and 77.

**Table 4:** Calculated (harmonic and anharmonic) and experimental normal vibrational frequencies of CHD_2_NO_3_ (in cm^–1^)

Mode		D-eclipsed		H-eclipsed		D-staggered		H-staggered		Expt.^*a*^
		*ω*_*i*_	*ν*_*i*_	*ω*_*i*_	*ν*_*i*_	*ω*_*i*_	*ν*_*i*_	*ω*_*i*_	*ν*_*i*_	*ν*_*i*_
1	*ν*_m_(CH)			3170	3020	3159	3010			3029
1	*ν*_⊥_(CH)	3135	2986					3120	2970	3014
2	*ν*_a_(CD_2_)	2368	2279	2344	2256	2353	2265	2375	2284	2300
3	*ν*_s_(CD_2_)	2251	2175	2248	2170	2246	2171	2256	2185	2194
4	*ν*_a_(NO_2_)	1624	1575	1628	1580	1626	1578	1622	1574	1574
5	*ν*_s_(NO_2_)	1421	1377	1420	1375	1420	1375	1421	1378	1388
6	*δ*∥(CH)	1313	1277	1316	1277	1315	1276	1304	1271	1283
7	*δ*_⊥_(CH)	1294	1258	1298	1266	1297	1267	1297	1258	1264
8	*δ*(CD_2_)	1076	1046	1060	1031	1065	1036	1080	1051	1057
9	*w*(CD_2_)	997	972	984	957	992	966	999	973	988
10	*ν*(CN)	912	890	957	937	937	916	908	886	895
11	*r*(CD_2_)	896	878	902	881	902	881	886	868	888
12	*δ*(NO_2_)	639	623	634	618	642	626	634	618	640
13	*w*(NO_2_)	578	568	560	551	556	547	591	581	559
14	*r*(NO_2_)	444	439	452	446	450	444	437	432	443

^*a*^Experimental data from ref. 79 and 81.

The computed harmonic fundamental frequencies are, in line with previous findings,^79^ found to be overestimated compared to experiment. Even with anharmonic corrections, the frequencies are in many cases overestimated compared to the experimental data, but lead to a significantly better agreement with experiment. The differences in the calculated vibrational frequencies for the different rotamers are in general very small. Indeed, very similar vibration frequencies are found for the two rotamers of CH_3_NO_2_ at both the harmonic and anharmonic level of calculation, the largest difference being 9 cm^–1^. The calculated vibrational frequencies are also in very good agreement with the experimental assignments of the modes.^80–83^

Experimentally, two peaks are assigned in the infrared spectrum to the stretching mode of the C–D bond in CH_2_DNO_2_: A strong mode at 2266 cm^–1^ corresponding to the stretching perpendicular to the plane of the nitro group, and a weak one at 2276 cm^–1^ corresponding to the stretching parallel to the same plane.^79^ We find that the maximum frequency for the *ν*(CD) mode is found for the D-eclipsed geometry in both the harmonic and anharmonic treatment, and the frequency decreases the further away the deuterium atom is from the NO_2_ plane.

A similar behaviour is also observed for the stretching of the CH bond in CHD_2_NO_2_, with a strong band at 3029 cm^–1^ corresponding to stretching perpendicular to the plane of the nitro group, and a weak band at 3014 cm^–1^ corresponding to stretching parallel to the same plane.^79^ The maximum frequency for the *ν*(CH) mode is found for the H-eclipsed geometry in both the harmonic and anharmonic treatment, and the frequency then decreases the further away the hydrogen atom is from the NO_2_ plane, in analogy to the observations for CH_2_DNO_2_.

#### 4.2.2 Infrared spectra

**Fig. 4:**
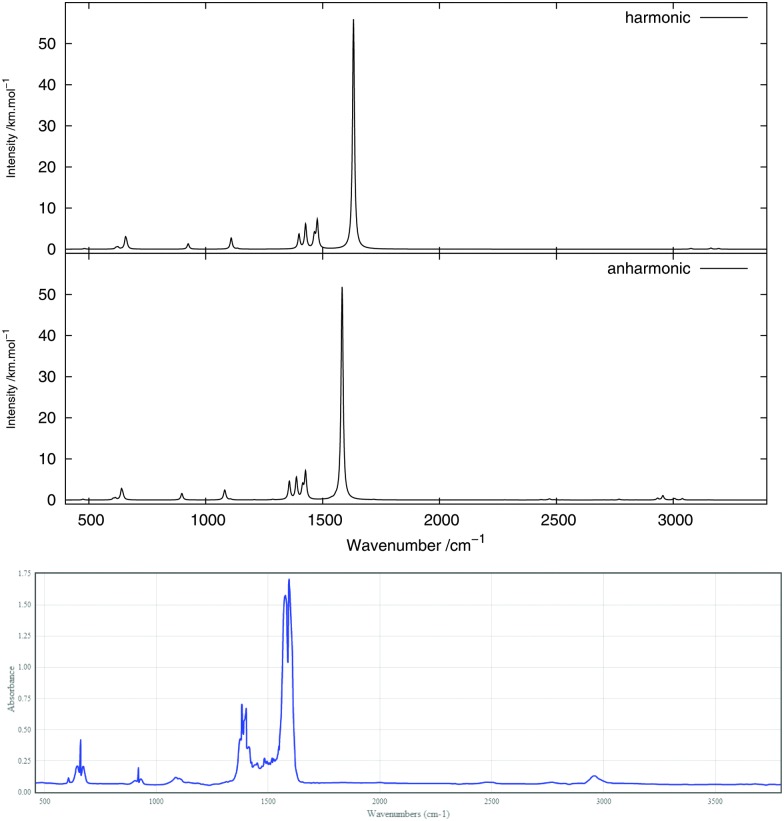
Infrared spectrum of CH_3_NO_2_: (top) harmonic; (middle) anharmonic; (bottom) experimental gas phase.^84^

**Fig. 5:**
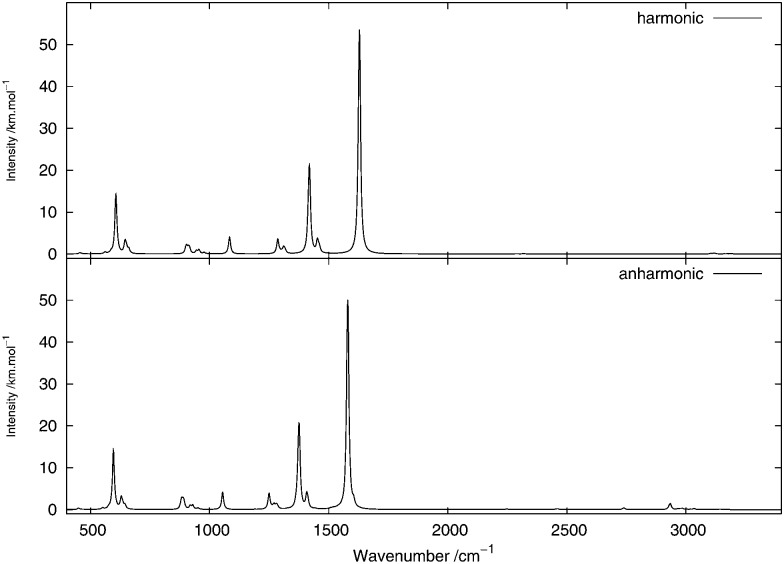
Infrared spectrum of CH_2_DNO_2_: (top) harmonic; (bottom) anharmonic.

**Fig. 6:**
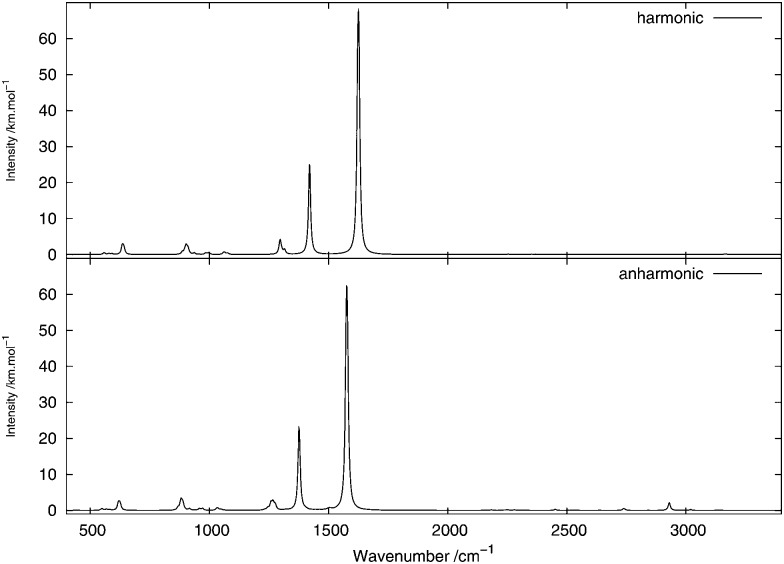
Infrared spectrum of CHD_2_NO_2_: (top) harmonic; (bottom) anharmonic.

The infrared spectrum is calculated by summing the calculated spectra of the three rotamers. The harmonic and anharmonic calculated infrared spectra for non-, mono- and di-deuterated isotopomers are shown in Fig. 4–6, respectively. Tables 5–7 show the calculated infrared spectral intensities (before Lorentzian normalization) for the normal modes of the non-, mono- and di-deuterated isotopomers.

**Table 5:** Calculated (harmonic and anharmonic) infrared spectral intensities of CH_3_NO_2_ (in km mol^–1^)

Mode		Eclipsed		Staggered	
		*I* IR harm	*I* IR anharm	*I* IR harm	*I* IR anharm
1	*ν*_a_(CH_3_)	2.15	3.94	0.57	1.29
2	*ν*_s_′(CH_3_)	1.78	2.63	2.25	3.14
3	*ν*_s_(CH_3_)	2.36	2.65	0.71	1.07
4	*ν*_a_(NO_2_)	580.62	550.36	298.07	251.14
5	*δ*_s_′(CH_3_)	94.00	92.23	11.30	11.33
6	*δ*_a_(CH_3_)	9.70	9.50	41.14	38.85
7	*ν*_s_(NO_2_)	38.31	40.69	54.95	41.37
8	*δ*_s_(CH_3_)	2.24	0.47	52.25	68.51
9	*ρ*_⊥_(CH_3_)	1.26	1.34	1.22	1.43
10	*ρ*_∥_(CH_3_)	30.97	30.22	11.83	8.53
11	*ν*(CN)	5.10	6.34	15.56	18.63
12	*δ*(NO_2_)	39.17	37.49	17.19	14.45
13	*ω*(NO_2_)	7.44	6.84	5.37	5.08
14	*ρ*(NO_2_)	1.76	1.90	0.82	0.97

**Table 6:** Calculated (harmonic and anharmonic) infrared spectral intensities of CH_2_DNO_2_ (in km mol^–1^)

Mode		D-eclipsed		H-eclipsed		D-staggered		H-staggered	
		*I* IR harm	*I* IR anharm	*I* IR harm	*I* IR anharm	*I* IR harm	*I* IR anharm	*I* IR harm	*I* IR anharm
1	*ν*_a_(CH_2_)	0.78	1.45	1.68	3.33	0.52	1.26	0.73	1.19
2	*ν*_s_(CH_2_)	0.27	0.71	1.13	1.85	2.55	3.50	2.06	2.36
3	*ν*_∥_(CD)	1.47	1.72					0.41	0.53
3	*ν*_m_(CD)			0.48	0.61	0.46	0.48		
4	*ν*_a_(NO_2_)	313.97	294.68	313.42	267.47	311.69	274.00	5.07	3.64
5	*δ*(CH_2_)	30.95	32.68	11.91	11.03	8.97	7.29	15.32	16.77
6	*ν*_s_(NO_2_)	100.44	89.24	99.15	97.13	98.87	96.26	86.22	66.26
7	*ω*(CH_2_)	4.65	3.47	7.35	8.87	11.06	13.08	8.14	7.37
8	*ρ*(CH_2_)	5.31	4.95	25.75	25.70	29.24	28.43	4.85	2.96
9	*δ*_⊥_(CD)	2.82	3.00	3.63	3.42	5.75	5.32	53.61	53.65
10	*δ*_∥_(CD)	11.45	12.49	5.27	5.23	0.84	0.75	14.51	15.02
11	*ν*(CN)	13.74	15.52	15.13	17.67	15.82	18.57	20.68	22.56
12	*δ*(NO_2_)	18.02	25.69	17.14	14.77	16.51	14.24	24.34	23.03
13	*ω*(NO_2_)	3.54	3.10	4.67	4.35	5.46	5.02	224.51	219.61
14	*ρ*(NO_2_)	0.32	0.39	0.85	0.95	0.74	0.88	4.88	4.97

**Table 7:** Calculated (harmonic and anharmonic) infrared spectral intensities of CHD_2_NO_3_ (in km mol^–1^)

Mode		D-eclipsed		H-eclipsed		D-staggered		H-staggered	
		*I* IR harm	*I* IR anharm	*I* IR harm	*I* IR anharm	*I* IR harm	*I* IR anharm	*I* IR harm	*I* IR anharm
1	*ν*_m_(CH)			2.26	3.29	1.45	0.55		
1	*ν*_⊥_(CH)	0.51	1.02					0.54	0.90
2	*ν*_a_(CD_2_)	1.29	1.47	0.79	0.75	1.38	0.73	0.71	1.07
3	*ν*_s_(CD_2_)	0.69	0.84	0.20	0.26	0.22	0.08	1.30	1.50
4	*ν*_a_(NO_2_)	325.55	304.44	318.48	290.41	320.01	291.90	328.88	310.06
5	*ν*_s_(NO_2_)	99.23	95.96	102.52	98.65	101.11	94.76	98.24	96.09
6	*δ*_∥_(CH)	3.62	3.90	8.99	9.12	7.01	7.51	8.00	8.52
7	*δ*_⊥_(CH)	19.83	18.52	15.38	13.99	19.61	13.44	10.98	10.85
8	*δ*(CD_2_)	3.35	2.37	8.05	7.63	6.23	6.26	2.56	2.57
9	*w*(CD_2_)	2.57	2.58	6.58	6.86	2.17	2.39	4.39	4.47
10	*ν*(CN)	14.22	16.22	0.48	0.47	6.69	6.74	15.79	18.02
11	*r*(CD_2_)	10.16	9.93	16.70	18.73	15.41	13.84	9.37	9.34
12	*δ*(NO_2_)	16.18	14.03	16.61	14.59	15.94	15.59	16.83	14.69
13	*w*(NO_2_)	4.75	4.42	4.21	3.85	4.98	4.76	3.83	3.44
14	*r*(NO_2_)	0.29	0.34	0.65	0.73	0.35	0.45	0.16	0.21

For the three isotopomers considered in this study, the anharmonic corrections do not substantially change the relative intensities of the different fundamental bands below 2000 cm^–1^. In this region, the main improvements arising from the anharmonic treatment is in the calculated vibrational frequencies, as discussed in the previous section. This observation is in agreement with the findings of Bloino and Barone^38^ using the GVPT2 approach with numerical calculation of the anharmonic IR spectra for a series of molecules.

For all three isotopomers, a weak feature appears in the anharmonic spectrum around 2940 cm^–1^ arising mainly from the combination of the symmetric (mode 4 for all isotopomers) and asymmetric (mode 7 for CH_3_NO_2_, mode 6 for CH_2_DNO_2_ and mode 5 for CHD_2_NO_2_) stretching modes of the NO_2_ fragment.

The experimental gas-phase spectrum of CH_3_NO_2_ from ref. 84 is reproduced in Fig. 4 for comparison. As already noted, a low-intensity feature around 3000 cm^–1^, also appearing in the experimental spectrum, is introduced with the anharmonic treatment. The main peak of this feature, at 2955 cm^–1^, arises mainly from the 4^1^7^1^ combination band and a minor contribution from the 3^1^ fundamental band. Apart from the 1^1^ and 2^1^ fundamental bands (3038 and 3005 cm^–1^, respectively), another low-intensity combination band, 4^1^8^1^ at 2933 cm^–1^, appears from the anharmonic treatment. Other low-intensity peaks, also present in the experimental spectrum, appear due to the 4^1^11^1^ combination band at 2471 cm^–1^ and the 7^2^ overtone band at 2768 cm^–1^.

In the CH_2_DNO_2_ spectrum, a small shoulder arising from the 10^1^12^1^ combination band of the H-eclipsed conformer and the 9^1^13^1^ combination band from the D-staggered conformer appears on the most intense peak. These two bands are combinations of an angular vibration of the NO_2_ fragment and an angular motion of the CD fragment. A low-intensity band appears from the anharmonic treatment around 3000 cm^–1^. The main peak of this band, around 2933 cm^–1^, corresponds to the 4^1^6^1^ combination band from all four conformers. The rest of the features of this band arise from the 1^1^ and 2^1^ fundamental bands of the four conformers. Other low-intensity peaks appear in the anharmonic spectrum around 2738 cm^–1^ due to the 6^2^ overtone band of the four conformers and around 2462 cm^–1^ due to the 4^1^11^1^ combination band from the H-eclipsed and D-staggered conformers.

In the CHD_2_NO_2_ spectrum, the anharmonic treatment gives a modification in the shape of the (broad) band between 1230 and 1280 cm^–1^, mainly due to the 6^1^ and 7^1^ fundamental bands and from the 12^2^ overtone band. A weak band appears around 1505 cm^–1^, due to the 10^1^12^1^ and 11^1^12^1^ combination bands from the four conformers. A low-intensity peak, corresponding to the 4^1^5^1^ combination band, appears around 2930 cm^–1^ from the anharmonic treatment. In addition to the peak at 3020 cm^–1^, corresponding to the 1^1^ fundamental band of the H-eclipsed conformer, another low-intensity peak appears in the anharmonic spectrum around 2740 cm^–1^ and is due to the 5^2^ overtone band.

#### 4.2.3 Raman spectra

**Fig. 7:**
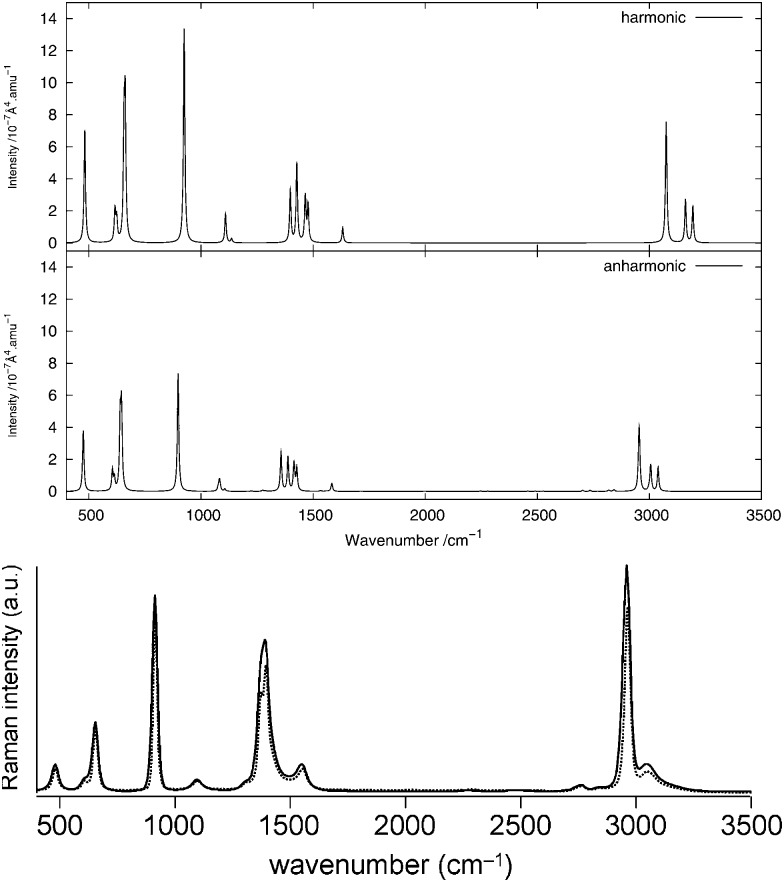
Raman spectrum of CH_3_NO_2_: (top) harmonic; (middle) anharmonic; (bottom) experimental liquid phase (measured with either a narrow-band laser (dotted curve) or an ultrafast laser (solid curve))^85^ (Adapted with permission from S. Shigeto *et al.*, *J. Phys. Chem. B*, 2008, **112**, 232. Copyright 2014 American Chemical Society.).

**Fig. 8:**
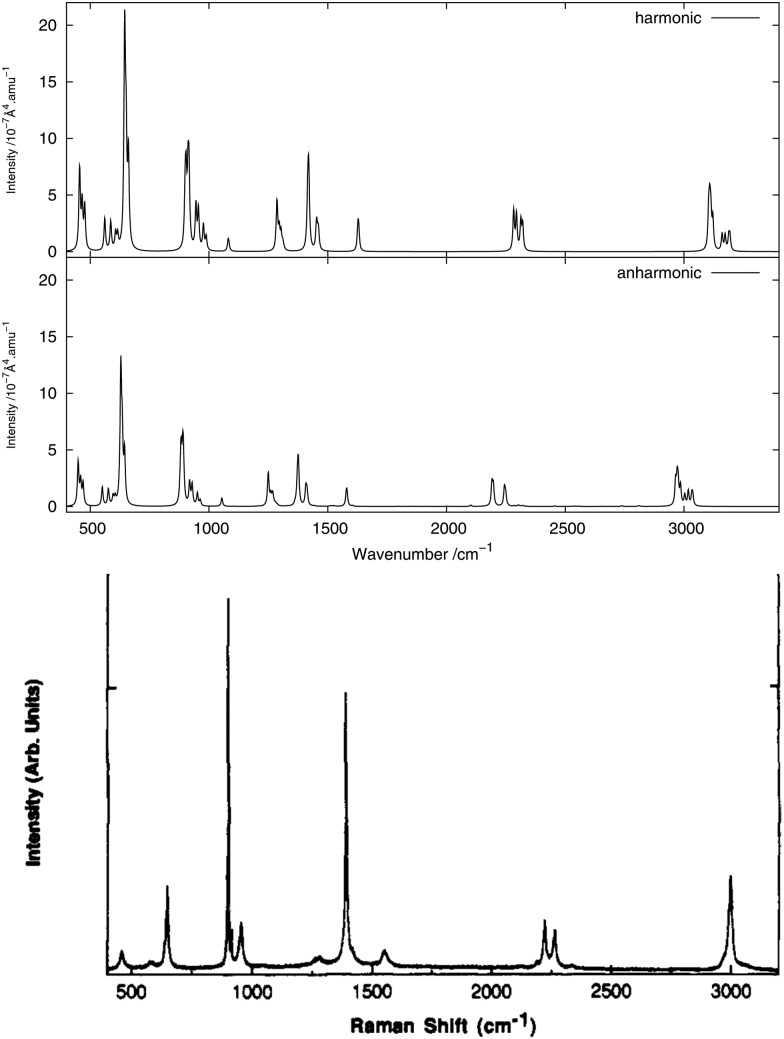
Raman spectrum of CH_2_DNO_2_: (top) harmonic; (middle) anharmonic; (bottom) experimental liquid phase^81^ (Adapted with permission from J. R. Hill *et al.*, *J. Phys. Chem.*, 1991, **95**, 3037. Copyright 2014 American Chemical Society.).

**Fig. 9:**
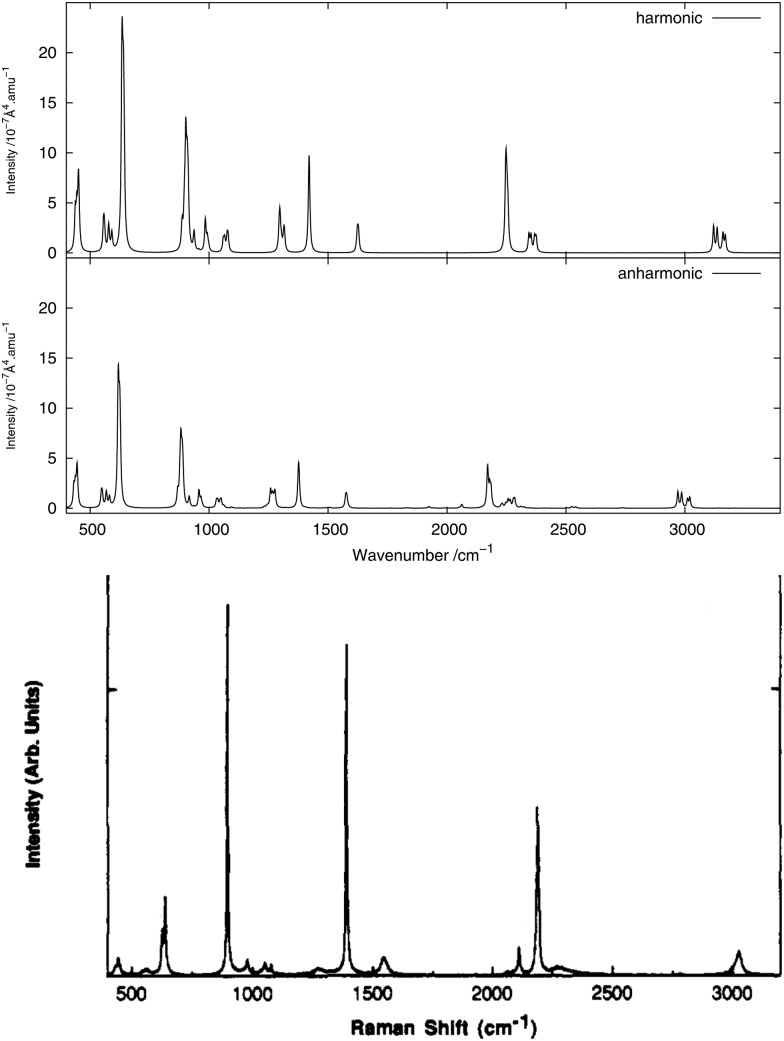
Raman spectrum of CHD_2_NO_2_: (top) harmonic; (middle) anharmonic; (bottom) experimental liquid phase^81^ (Adapted with permission from J. R. Hill *et al.*, *J. Phys. Chem.*, 1991, **95**, 3037. Copyright 2014 American Chemical Society.).

As done for the IR spectra, the Raman spectra were obtained as the sums of Raman spectra for the individual rotamers. The calculated harmonic and anharmonic Raman spectra for non-, mono- and di-deuterated isotopomers are shown in Fig. 7–9, respectively. Tables 8–10 show the calculated Raman spectral intensities (before Lorentzian normalization) for the normal modes of the non-, mono- and di-deuterated isotopomers.

**Table 8:** Calculated (harmonic and anharmonic) Raman spectral intensities of CH_3_NO_2_ (in 10^–7^ Å^4^ amu^–1^)

Mode		Eclipsed		Staggered	
		*I* Raman harm	*I* Raman anharm	*I* Raman harm	*I* Raman anharm
1	*ν*_a_(CH_3_)	12.28	8.82	14.39	9.08
2	*ν*_s_′(CH_3_)	15.44	9.79	16.63	10.46
3	*ν*_s_(CH_3_)	45.08	26.05	48.86	28.04
4	*ν*_a_(NO_2_)	2.70	1.52	8.59	4.07
5	*δ*_s_′(CH_3_)	13.28	8.23	13.64	8.29
6	*δ*_a_(CH_3_)	13.32	8.18	22.46	13.76
7	*ν*_s_(NO_2_)	36.42	15.18	22.43	10.03
8	*δ*_s_(CH_3_)	26.02	19.08	12.88	10.09
9	*ρ*_⊥_(CH_3_)	0.81	0.42	2.23	1.32
10	*ρ*_∥_(CH_3_)	8.16	4.78	13.38	5.70
11	*ν*(CN)	75.59	41.59	82.64	45.52
12	*δ*(NO_2_)	76.91	41.88	94.92	58.54
13	*ω*(NO_2_)	16.84	8.74	23.15	14.94
14	*ρ*(NO_2_)	39.01	20.90	44.62	23.87

**Table 9:** Calculated (harmonic and anharmonic) Raman spectral intensities of CH_2_DNO_2_ (in 10^–7^ Å^4^ amu^–1^)

Mode		D-eclipsed		H-eclipsed		D-staggered		H-staggered	
		*I* Raman harm	*I* Raman anharm	*I* Raman harm	*I* Raman anharm	*I* Raman harm	*I* Raman anharm	*I* Raman harm	*I* Raman anharm
1	*ν*_a_(CH_2_)	17.38	10.95	14.33	11.58	14.42	9.06	16.05	15.50
2	*ν*_s_(CH_2_)	39.30	24.11	33.83	21.09	30.68	19.30	37.84	23.54
3	*ν*_∥_(CD)	26.10	10.35					26.62	15.74
3	*ν*_m_(CD)			36.97	20.53	40.73	22.41		
4	*ν*_a_(NO_2_)	10.43	5.79	10.19	5.10	9.98	5.16	10.43	5.69
5	*δ*(CH_2_)	15.86	9.59	13.27	7.94	9.88	5.70	15.83	9.59
6	*ν*_s_(NO_2_)	37.16	18.43	28.95	14.77	31.04	16.01	31.79	13.19
7	*ω*(CH_2_)	0.49	0.28	2.80	2.19	4.88	4.24	0.95	0.63
8	*ρ*(CH_2_)	17.36	10.27	26.72	16.03	26.15	15.76	20.80	9.88
9	*δ*_⊥_(CD)	0.62	0.41	5.40	3.08	7.33	3.82	2.25	1.42
10	*δ*_∥_(CD)	45.65	23.57	26.09	12.92	13.89	5.93	41.00	21.04
11	*ν*(CN)	55.33	32.47	64.80	37.43	72.07	40.31	56.78	33.44
12	*δ*(NO_2_)	110.63	69.01	97.53	60.92	93.14	49.61	109.35	68.26
13	*ω*(NO_2_)	14.64	7.61	29.55	16.98	33.24	19.38	16.41	9.04
14	*ρ*(NO_2_)	41.38	22.13	46.31	24.74	44.23	22.61	43.71	23.29

**Table 10:** Calculated (harmonic and anharmonic) Raman spectral intensities of CHD_2_NO_3_ (in 10^–7^ Å^4^ amu^–1^)

Mode		D-eclipsed		H-eclipsed		D-staggered		H-staggered	
		*I* Raman harm	*I* Raman anharm	*I* Raman harm	*I* Raman anharm	*I* Raman harm	*I* Raman anharm	*I* Raman harm	*I* Raman anharm
1	*ν*_m_(CH)			18.97	12.05	21.72	9.88		
1	*ν*_⊥_(CH)	27.53	17.22					30.50	19.09
2	*ν*_a_(CD_2_)	16.84	8.27	20.23	8.76	18.52	7.83	16.57	9.40
3	*ν*_s_(CD_2_)	43.57	20.69	51.09	28.41	49.17	19.58	40.04	19.45
4	*ν*_a_(NO_2_)	11.94	6.57	10.84	5.78	11.11	6.00	12.45	6.91
5	*ν*_s_(NO_2_)	29.33	14.92	30.66	15.65	30.13	15.41	29.04	14.93
6	*δ*_∥_(CH)	9.31	4.98	12.67	7.63	11.60	6.43	9.89	5.89
7	*δ*_⊥_(CH)	17.97	10.90	10.29	5.95	13.27	6.31	15.56	9.40
8	*δ*(CD_2_)	15.39	5.97	13.87	8.04	13.67	7.65	16.12	8.39
9	*w*(CD_2_)	3.33	1.27	36.96	19.83	14.72	9.82	2.19	0.74
10	*ν*(CN)	57.75	32.21	1.66	1.02	22.31	11.96	70.13	39.80
11	*r*(CD_2_)	36.87	20.89	59.95	34.08	59.59	28.80	27.88	15.85
12	*δ*(NO_2_)	103.21	63.69	109.73	68.89	105.29	58.31	110.54	68.81
13	*w*(NO_2_)	30.03	17.50	24.79	13.01	28.92	14.78	20.69	11.26
14	*r*(NO_2_)	41.56	22.22	47.91	25.45	43.99	23.64	41.98	22.41

The relative intensities of the bands corresponding to CH or CD vibrations compare rather well with the experimental data, as well as the relative intensities of the bands corresponding to the CN and NO_2_ motions. However, the agreement between theory and experiment for the relative intensities of these two different vibrations is poor. The anharmonic treatment gives slightly better agreement with experiment, though the differences are very small.

The major correction arising from the anharmonic treatment occurs for the frequencies, as also noted for the IR spectra. Anharmonic corrections do not modify significantly the relative intensities of the fundamental bands, except for the band at 1380 cm^–1^ corresponding to the ν_s_(NO_2_) mode that is weakened by the anharmonic treatment for all three isotopomers relative to the harmonic model. However, for both of the deuterated isotopomers, a slight improvement in the intensities of the band corresponding to the 3^1^ mode for the mono-deuterated and the 2^1^ mode for the di-deuterated isotopomer (both corresponding to a stretching of the CD bonds) seems to appear from the anharmonic treatment, where they are reduced slightly more than the other bands that correspond to hydrogen-related motions. This modification of the relative intensities leads to somewhat better agreement with experiment.

We note that the liquid-phase IR spectrum for CH_3_NO_2_ (as can be seen in Fig. 1(a) of ref. 85, not reproduced here) presents strikingly different spectral intensities to the gas-phase spectrum. Therefore, a corresponding effect can be expected also for the Raman spectra. This could explain at least some of the differences between the calculated (gas-phase) and experimental (liquid-phase) spectra presented here, and solvent effects will be investigated in future work.

The experimental liquid-phase spectrum of CH_3_NO_2_ from ref. 85 is reproduced in Fig. 7 for comparison. A weak peak appears around 1280 cm^–1^ corresponding to the 12^2^ overtone. The small shoulder appearing in the experimental spectrum on the low-frequency side of the peak around 1400 cm^–1^ could be related to this overtone band. Other very weak peaks appear between 2700 cm^–1^ and 2850 cm^–1^ and are due to several different overtones and combination bands. These features could be related to the weak peak appearing in the experimental spectrum around the same frequency. A large number of features in the theoretical spectrum appear between 1600 and 2900 cm^–1^ and arise from the contributions of different combination and overtone bands.

The experimental liquid-phase spectrum of CH_2_DNO_2_ from ref. 81 is reproduced in Fig. 8 for comparison. A major improvement in comparison to the experimental spectrum is observed when including anharmonic corrections for the splitting of the 3^1^ band with respect to the position of the deuterium atom, around 2195 cm^–1^ if the CD bond stretches almost parallel to the NO_2_ plane and around 2295 cm^–1^ if it stretches almost perpendicular to it, as seen in the experimental spectrum. A weak peak appears in the anharmonic spectrum around 2250 cm^–1^, corresponding to the 6^1^11^1^ combination band, mainly from the D-eclipsed rotamer. Another weak feature appears around 2310 cm^–1^, corresponding to the 8^1^9^1^ combination band present in all the four rotamers.

The experimental liquid-phase spectrum of CHD_2_NO_2_ from ref. 81 is reproduced in Fig. 9 for comparison. The band between 2250 and 2290 cm^–1^, arising from the 2^1^ mode in the harmonic treatment, is broadened in the anharmonic spectrum, mainly due to the 6^1^9^1^ combination of the four rotamers on the low-frequency side and to the 6^1^8^1^ combination bands on the high-frequency side of the D-eclipsed and H-eclipsed rotamers. Two weak peaks appear around 1920 and 2060 cm^–1^ due, respectively, to the 8^2^ and 9^2^ overtones, primarily from the D-staggered rotamer. A very weak band also appears around 2530 cm^–1^, corresponding to the 6^2^ and 7^2^ overtones, mainly from the D-staggered rotamer.

## 5 Summary and concluding remarks

We have presented the first analytic calculations of anharmonic corrections to both the vibrational frequencies and intensities in infrared and Raman spectroscopies. This has been made possible by our recent development of a recursive scheme for the calculation of high-order molecular properties, including properties involving frequency-dependent perturbations and perturbation dependence in the basis set.^47^ The approach is applicable to single-determinant self-consistent field models such as Hartree–Fock theory and Kohn–Sham DFT, and being matrix-based, it can also be extended to linear-scaling approaches,^48,62^ as well as to the relativistic four-component level of theory.^86^ We have previously applied our approach to the calculation of anharmonic corrections to vibrational frequencies using density functional theory^46^ and to the analytic calculation of Raman optical activity^87^ and hyper-Raman scattering,^71^ and in this work, we have used it to calculate anharmonic infrared and Raman spectra of nitromethane and its partially deuterated isotopomers. We find that anharmonic effects lead to an improvement in the quality of the computed IR and Raman spectra. The major improvements arising from the anharmonic treatment occur for the vibrational frequencies, while the effects of the anharmonic corrections on the infrared and Raman intensities are smaller and show only minor influences on the relative intensities of the fundamental bands, which is in agreement with earlier observations.^38^ Nevertheless, the anharmonic corrections are important in order to capture the overtone and combination bands. The anharmonic corrections are found to be somewhat more important for the Raman spectra, even if very small, than for the infrared spectra. Overall, the anharmonic spectra are in better agreement with experiment than the corresponding harmonic spectra.

We have also shown that evaluating the energy and property derivatives by numerical differentiation is prone to numerical instabilities, as also noted elsewhere,^51^ so that obtaining reliable numerical derivatives can prove difficult for general molecular systems, and we have seen that the errors thus introduced can significantly affect the calculated results, whereas analytic approaches would be free of these sources of error. Because of this, we believe that analytical derivatives of high order is an important step in making the inclusion of anharmonic corrections in calculated infrared and Raman spectra routine, leading to an improved understanding of the importance and occurrence of anharmonic effects in vibrational spectroscopies.

Finally, solvent effects are known to affect vibrational spectroscopies.^88^ It is therefore important that the scheme presented here is extended to include solvent effects, either in the form of polarizable continuum models or polarizable embedding approaches,^89^ and work in this direction is in progress.^90^
